# Supplementary material for: Pseudotyped Bat Coronavirus RaTG13 is efficiently neutralised by convalescent sera from SARS-CoV-2 infected patients
Source: Commun Biol. 2022 May 3;5:409. doi: 10.1038/s42003-022-03325-9 (PMC9065041; doi:10.1038/s42003-022-03325-9)
Supplement: Supplementary file 2 — Supplementary Information [file 42003_2022_3325_MOESM2_ESM.pdf]

Supplemental Figure 1

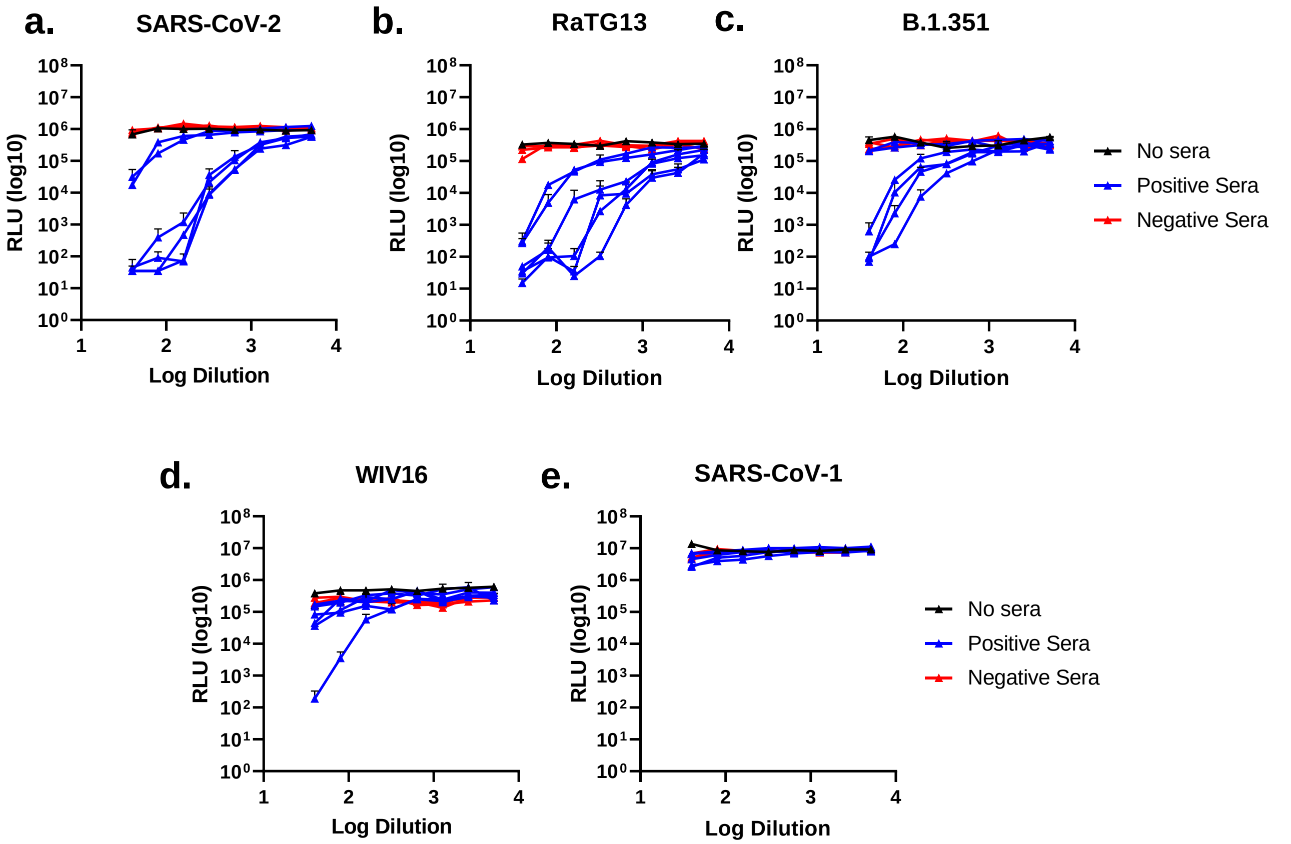

Supplementary Figure 1: Exemplar neutralisation data from Figure 1B, showing the raw relative light units (RLU) from pseudotyped virus neutralization assays (pMNs). Serial dilutions of convalescent sera obtained from SARS-CoV-2 infected individuals during the first wave of the SARS-CoV-2 pandemic in the UK were used. For each pseudotype (a) SARS-CoV-2, (b) RaTG13, (c) B.1.351/Beta, (d) WIV16 and (e) SARS-CoV-1 we show five SARS-CoV-2 positive sera samples (blue lines), two confirmed seronegative sera samples (red lines), and a singular no-pseudovirus controls (black line), representative of a selection of data shown in Figure 1b. IC50s for each sample were calculated as previously described in Ferrara et al, 2018. This assay workflow was used for all pMNs throughout the study.

Supplemental Figure 2

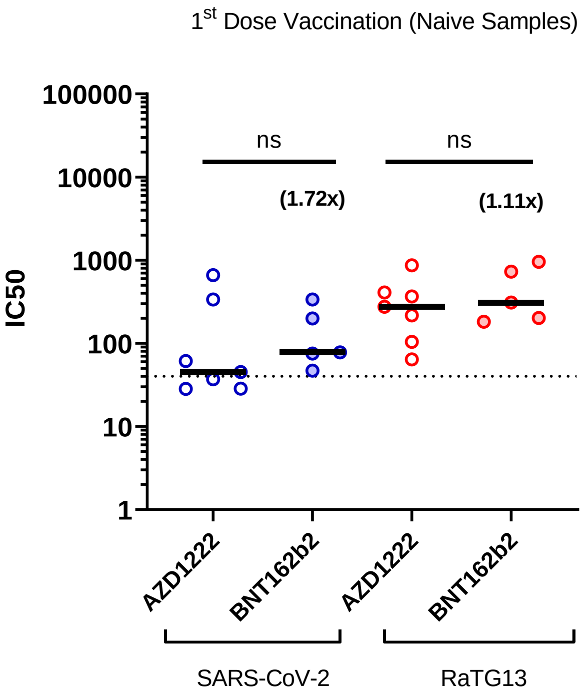

Supplemental Figure 2: Differences in neutralisation potencies after a single dose of AZD1222 (n=9) or BNT162b2 (n=12) in infection-naïve serum samples against either SARS-CoV-2 or RaTG13 (data sub-divided from Figure 1c and 1d). No significant difference in neutralisation titres between vaccines, against either SARS-CoV-2 (p=0.26) or RaTG13 (p=0.63), were detected.

Supplemental Figure 3

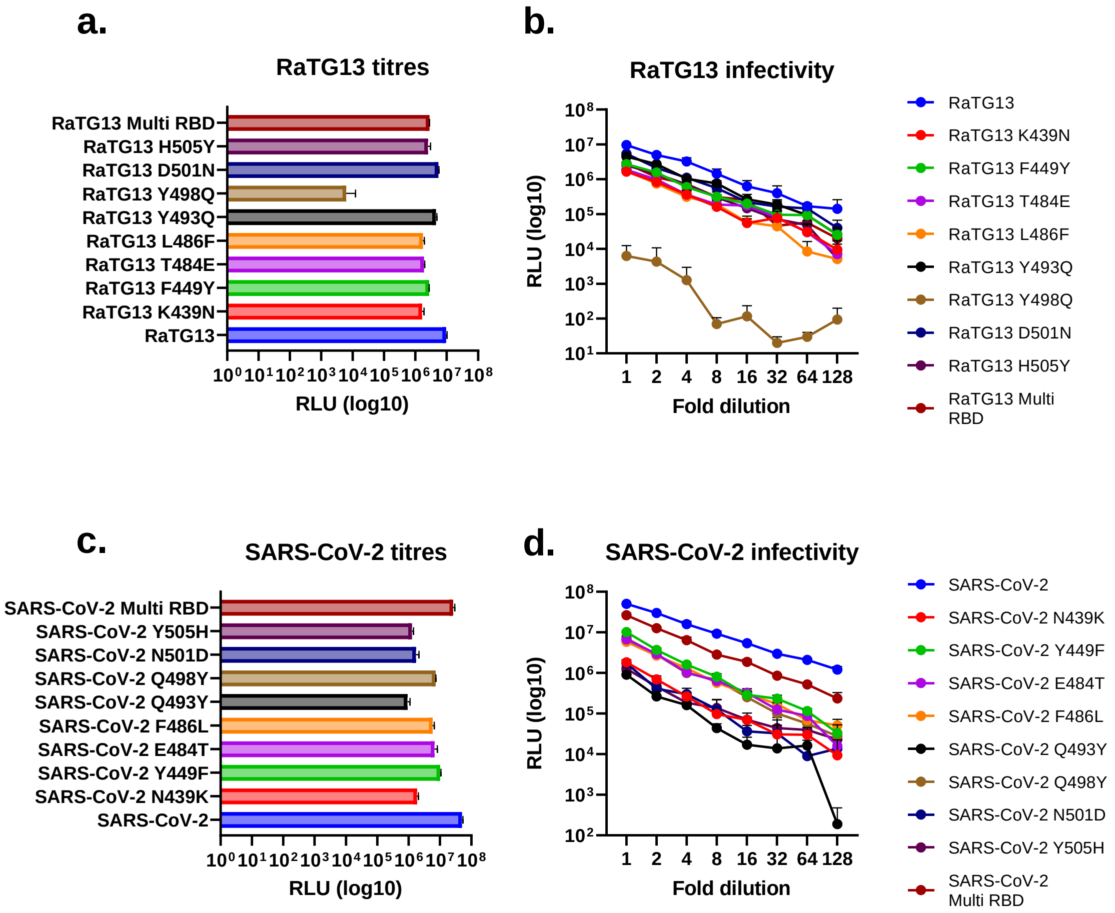

Supplemental Figure 3: Characterisation of pseudoparticle infectivity of all RaTG13 (a, b) and SARS-CoV-2 (c, d) viruses and mutants. We were unable to rescue sufficient titres of RaTG13 Y498Q (a), therefore neutralisation experiments using this mutant were not carried out in Figure 2d. The Q493Y substitution in SARS-CoV-2 Spike caused the greatest relative reduction in infectivity (b).
