# Supplementary material for: Pseudotyped Bat Coronavirus RaTG13 is efficiently neutralised by convalescent sera from SARS-CoV-2 infected patients
Source: Commun Biol. 2022 May 3;5:409. doi: 10.1038/s42003-022-03325-9 (PMC9065041; doi:10.1038/s42003-022-03325-9)
Supplement: Supplementary file 3 — Description of Additional Supplementary Files [file 42003_2022_3325_MOESM3_ESM.pdf]

Description of Additional Supplementary Files

File name: Supplementary Data 1
Description: Source data for manuscript.
